# Chia (*Salvia hispanica* L.), a Pre-Hispanic Food in the Treatment of Diabetes Mellitus: Hypoglycemic, Antioxidant, Anti-Inflammatory, and Inhibitory Properties of α-Glucosidase and α-Amylase, and in the Prevention of Cardiovascular Disease

**DOI:** 10.3390/molecules28248069

**Published:** 2023-12-13

**Authors:** Rosario Tavera-Hernández, Manuel Jiménez-Estrada, J. Javier Alvarado-Sansininea, Maira Huerta-Reyes

**Affiliations:** 1 Instituto de Química, Universidad Nacional Autónoma de México, Ciudad Universitaria, Coyoacán, Ciudad de México 04510, Mexico; rosario.tavera@gmail.com (R.T.-H.); manueljemex@gmail.com (M.J.-E.); 2 Herbario FEZA, Facultad de Estudios Superiores Zaragoza, Universidad Nacional Autónoma de México, Batalla de 5 de mayo S/N, Col. Ejército de Oriente, Ciudad de México 09230, Mexico; javier33@comunidad.unam.mx; 3 Unidad de Investigación Médica en Enfermedades Nefrológicas, Hospital de Especialidades “Dr. Bernardo Sepúlveda Gutiérrez”, Centro Médico Nacional Siglo XXI, Instituto Mexicano del Seguro Social, Cuauhtémoc, Ciudad de México 06720, Mexico

**Keywords:** chia, diabetes mellitus, *Salvia hispanica*, hypoglycemic, antioxidant, anti-inflammatory, antidiabetic, α-glucosidase, α-amylase, cardiovascular disease

## Abstract

Diabetes mellitus (DM) is considered one of the major health diseases worldwide, one that requires immediate alternatives to allow treatments for DM to be more effective and less costly for patients and also for health-care systems. Recent approaches propose treatments for DM based on that; in addition to focusing on reducing hyperglycemia, they also consider multitargets, as in the case of plants. Among these, we find the plant known as chia to be highlighted, a crop native to Mexico and one cultivated in Mesoamerica from pre-Hispanic times. The present work contributes to the review of the antidiabetic effects of chia for the treatment of DM. The antidiabetic effects of chia are effective in different mechanisms involved in the complex pathogenesis of DM, including hypoglycemic, antioxidant, and anti-inflammatory mechanisms, and the inhibition of the enzymes α-glucosidase and α-amylase, as well as in the prevention of the risk of cardiovascular disease. The tests reviewed included 16 in vivo assays on rodent models, 13 clinical trials, and 4 in vitro tests. Furthermore, chia represents advantages over other natural products due to its availability and its acceptance and, in addition, as a component of the daily diet worldwide, especially due to its omega-3 fatty acids and its high concentration of dietary fiber. Thus, chia in the present work represents a source of antidiabetic agents that would perhaps be useful in novel clinical treatments.

## 1. Introduction

DM is considered the most common metabolic disorder at present, in which the number of cases worldwide in the year 2000 exceeded 171 million, with an estimated projection for the year 2023 of this case number being duplicated [1]. Persons with diabetes represent a high cost to health-care systems worldwide, since they use medications, require periodic laboratory controls, and may have the need for long-term hospitalization care; in addition, the disease also leads to severe complications that can be disabling, such as amputations, blindness, and renal and cardiovascular diseases; furthermore, in some countries such as the U.S., it has been calculated that individuals living with DM spend 2.5 times more on medical assistance than persons without the disease [2]. Consequently, the cost of DM worldwide requires immediate alternatives that permit the treatment of DM to be more effective and less costly for persons living with DM and, in addition, less expensive for health-care systems.

Due to DM being a complex and multifactorial disease that affects all systems in the body, it is a disease in which, in addition, it is observed that its close pathophysiological correlation with obesity, inflammation, and insulin resistance generates a highly complicated disease to manage. Thus, recent approaches propose an integrative treatment for DM instead of focusing on reducing hyperglycemia, because the chronicity and systemic complications of DM compromise the long-term effectiveness of this reductionist treatment. In this manner, treatments that can target multiple organ systems or the physiological processes in the glucose homeostasis and pathophysiology of diabetes would be an alternative [3]. In this regard, a number of investigations report that plants have contributed valuable multitarget drugs that have been crucial in the management of several diseases, including chronic diseases such as diabetes, cancer, and neurological and hepatic afflictions [4]. Therefore, the relevance of reviewing information on plants that could be a potential source of multitarget drugs for the treatment of DM is imperative. Among these plants, we find that the plant popularly known as chia is highlighted, not only because of its reported multitarget action but also due to its being a highly nutrient-dense food that provides maximal health benefits to humans. 

The scientific name for chia is *Salvia hispanica* L. and it belongs to the Lamiaceae family [5]. Chia is a crop that has been cultivated since the year 3500 BC in Mesoamerica. It is a plant native to Mexico and Guatemala that, from pre-Hispanic times, formed an important part of the diet in the Aztec and Maya cultures, and it continues to be widely consumed in Mexico to date. Together with corn, beans, and amaranth, chia represented the crucial crops that conformed the basic diet of ancient populations in America [6]. Chia seeds comprised the most used part of the plant in culinary and medicinal uses to a greater extent than the leaves, which recently have attracted attention due to their chemical and nutritional composition. As part of beverages and as a component of food dishes, chia seeds have been employed from ancient times to the present and currently, in Mexico, a fresh beverage is prepared with chia seeds, water, and sugar, while, in some central and southern regions, these seeds are also mixed with cucumber and lime. As a component of food dishes, in pre-Columbian times, chia seeds were mixed with corn kernels to obtain a flour known as *chianpinolli*, which was employed to prepare tortillas and tamales [7,8]. With respect to traditional medicinal uses, among the most frequent of these in terms of chia, we find those for the treatment of skin conditions, gastrointestinal diseases, and ophthalmologic affectations [7]. Nevertheless, over the last decades, chia has been constantly studied around the world because of its nutritional contents and benefits to human health. Chia has been recognized as an excellent source of omega-3 fatty acids, of antioxidants, and for its high concentration of dietary fiber. Chia seeds are characterized by their oil content, which is approximately 25–40%, comprising around 60% of omega-3 alpha-linolenic acid (ALA) and 20% of omega-6 linolenic acid. The protein composition of chia seeds falls within a range of 15–25%, fats 30–33%, carbohydrates between 26 and 41%, fiber 18–30%, and ash approximately 4–5%, while minerals, vitamins, and antioxidants (such as chlorogenic acid, caffeic acid, myricetin, quercetin, and kaempferol) are present in elevated amounts [9,10]. Currently, chia has become very popular worldwide, forming part of daily menus, and is found in salads, bakery products, beverages, sausages, flours, pasta, and sweets. The number and variety of health benefits that the consumption of chia seeds has exhibited can permit the consideration of chia as “the seed for the first 21st century” [9,11]. Therefore, the aim of this review was to assess the effect of chia on different metabolic processes involved in the pathogeny of DM to a greater degree than solely its hypoglycemic effect but also the antioxidant and anti-inflammatory effects and in the inhibition of the enzymes α-glucosidase and α-amylase, as well as in the prevention of the risk of cardiovascular disease. The latter have been cited on review of the currently available literature and in an attempt to highlight the evidence shown by clinical trials and in vivo studies on rodents, in which chia, due to its nutrimental content, would be useful as part of a safe daily diet for the diabetic population. 

## 2. Chemical and Nutritional Composition of Chia 

Chia seeds are recognized for their nutritional and beneficial health properties. Chia is distinguished by its high carbohydrate (~43%), fiber (~34%), fat (~31%), and protein (~16%) content. Table 1 presents the nutritional profile per 100 g of chia seeds, according to that reported by the United States Drug Administration (USDA) National Nutrient Database for Standard Reference (SR) [12]. The fatty acid profile of chia seeds indicates a high content of polyunsaturated fatty acids (23%), among these linolenic acid (C18:3-ω-3) and linoleic acid (C18:2-ω-6) [13]. In addition, the seed is an excellent source of minerals, an even better source of these than other cereals such as rice, corn, wheat, quinoa, and amaranth; the most abundant minerals found in chia seeds are phosphorus, calcium, potassium, and magnesium [14]. On the other hand, chia seeds are not considered a good source of vitamins [15,16], although they possess a higher amount of thiamine (B1), riboflavin (B2), and niacin (B3) than rice, corn, wheat, quinoa, and amaranth [14,17].

### 2.1. Proteins

Protein content in chia seed is approximately 16.5%, according to that reported in the National Nutrient Database (SR) for 2018. However, some factors, such as climate and agrochemical conditions, can modify this value [22], in particular, because of the close relation between the temperature and the altitude where the chia seed grows. Hot temperatures favor protein synthesis and, consequently, a greater protein content [23,24]; for example, da Silva reported that the protein content of chia grown in Brazil was about 19% [15], while Jin et al., reported a content of 24.2% [18] and, in the Silveira et al., report, this was 19.6% [19]. 

The main protein fraction of chia seeds includes globulins (52%), followed by albumins (17%), glutelins (14%), and prolamins (17%) [25]. Chia seed proteins demonstrate good digestibility (78.9%), similar to that of casein (88.6%); this value is an indicator of their susceptibility to proteolytic enzymes [26]. The most abundant amino acids are glutamic acid (123 g/kg raw protein or 2.87–3.5 g/100 g of chia seed), arginine (80.6 g/kg raw protein or 2.0–2.14 g/100 g of chia seed), and aspartic acid (61.3 g/kg raw protein or 1.28–1.69 g/100 g of chia seed). Due to the content and the nature of the proteins, chia has been suggested as an attractive additive in the preparation of bakery products and emulsions [20] and as an ingredient to improve the protein content of a food [27]. Additionally, from the nutraceutical point of view, the glutamic acid content in chia stimulates the central nervous and immune systems in humans, while aspartic acid contributes to hormone regulation for nervous system function [25]. 

### 2.2. Fiber 

Chia seeds exhibit a total carbohydrate content within a range of 17–42% and a total dietary fiber between 23 and 34% [12]. Fiber values in chia seed differ according to the country of origin: in Mexico, total fiber is 34.5% [20]; in Guatemala, this is about 19% [28]; and, in Brazil, this is reported as about 23.7% [19] or even with highest values within a range of 33.37–37.18% [15]. This fiber is a mixture of carbohydrate polymers, including oligosaccharides and polysaccharides such as cellulose, pectin, and gums associated with lignin and other components [29,30]. Total dietary fiber is important in the diet due to its gel-forming properties that absorb water, which interacts with the polar groups of carbohydrates, in turn, increasing peristalsis [31]. The content of total dietary fiber (TDF) in chia grown in the state of Jalisco, Mexico, is 39.94 g/100 g on a dry-weight basis, and soluble dietary fiber (SDF) and insoluble dietary fiber (IDF) are 6.84 and 34.9 g/100 g, respectively [32]. The American Dietetic Association (ADA) recommends ingesting between 25 and 30 g/day of fiber, with an IDF/SDF ratio of 3-1 [30]. According to these latter recommended values, the Mexican chia seed possesses a higher fiber content, thus representing a good option for its inclusion in the diet in adequate amounts. IDF is the majority portion of TDF, and the main component of IDF is denominated the Klason lignin, followed by cellulose, hemicellulose, and uronic acid, which have been identified in lower amounts [32]. TSDF is formed from the neutral sugars that become evident when the seed is placed into water, forming a mucilage. This mucilage is formed mainly by repeating units of the tetra saccharide (→4)-β-D-xylopuranose-(1 → 4)α-glucopyranose-(1→)-β-D-xylopyranose with 4-O-methyl-α-D-glucuronic-acid branches in O-2 of the β-D-xylopyranose residue; this polysaccharide entertains a high capacity for forming a gel that traps water and fat [33]. The acid hydrolysis of the mucilage generates β-D-xylose, α-D-glucose, and -O-methyl-α-D-glucuronic acid at a 2:1:1 ratio, respectively [30].

### 2.3. Fats 

The amount of total fats in the chia seed has been reported to be between 30 and 40% [17,18,19], and this value and the fatty acid content depend on the environmental conditions of the plant’s place of origin and also on the analytical method employed for their determination [13,34,35]. Furthermore, the high content of polyunsaturated fatty acids in chia seed could produce lipid oxidation and a decrease in nutritional value during storage [36]. Between 23 and 79% of total fat is constituted of polyunsaturated fatty acids (PUFA), such as linolenic acid (18:3, ω-3) with 16–63% and linoleic acid (C18:2, ω-6) with 5–18%. There are also some monounsaturated fatty acids at a lower proportion (2–10%), among which oleic acid (18:1, ω-9) is highlighted with 2–10%; other monounsaturated fatty acids found at a lesser proportion include palmitoleic acid (C16:1) and *cis*-eicosadienoic acid (C20:2, ω-6). Meanwhile, the total content of saturated fatty acids is between 3 and 10%, with palmitic acid (C16:0) at a great proportion with 2–7% and stearic acid (C18:0) with an abundance of 0.91–3% [12]. As mentioned previously, the content of fatty acids can vary slightly depending on the place of origin and, in comparison with certain cereals, the chia seed has a higher content of linolenic acid than rice, corn, wheat, quinoa, and amaranth [14]. This is important because PUFA are essential for humans, in that they are not synthesized and must be incorporated into the diet [37]; in addition, ω-3 fatty acids possess beneficial effects, due to the fact that they favor the reduction in high levels of cholesterol, possess anti-inflammatory, cardioprotective, and hepatoprotective activities, and have also exhibited antidiabetic action and protection against cancer and arthritis. Meanwhile, ω-6 fatty acids exhibit anti-inflammatory, antihypertensive, and anticancer activity [14].

Diverse studies show that poly- and monounsaturated fatty acids could improve the glycemic level in patients with DMT2 [38]. Specifically, linoleic acid diminished the risk of developing DMT2 and inhibited the protein tyrosine phosphatases associated with insulin resistance; these proteins are considered targets for treating DMT2 [39,40]. Oleic acid stimulates glucose uptake into adipocytes by the phosphorylation of the insulin receptor [41] and by regulating the genes related to the PI3K signaling pathway, which regulate insulin sensitivity in the adipocytes [42]; furthermore, oleic acid prevents the inflammation and insulin resistance produced by palmitic acid in adipose tissue, suggesting that oleic acid possesses a similar protective effect to that of metformin against palmitic acid effects [43]. 

### 2.4. Secondary Metabolites

In addition to the previously mentioned primary metabolites, some secondary metabolites have also been detected in chia seeds, mainly compounds belonging to the group of polyphenols that are found free or bound to sugars [32,44,45].

The content of phenolic compounds has been reported as ranging between 641.7 and 921.1 µg/g of chia seed extract expressed as gallic acid equivalents (GAE) [19,32]. The following phenolic acids have been identified in chia seeds: gallic acid; caffeic acid; chlorogenic acid; protocatechuic ethyl ester; rosmarinic acid; rosmarinic acid glucoside and dihydroxybenzoyl glucoside; vanillic acid glucoside; and salvianic acid A and salvianolic acid B/E, in addition to the phenols oresbiusin A and dihydroxybenzaldehyde; the flavonoids apigenin, kaempferol, quercetin, and myricetin; the isoflavonoids daidzein, glycitein, genistein, and genistin; and the catechin derivative, epicatechin. Table 2 depicts the secondary metabolites and their concentrations identified in various investigations, where values range widely depending of the place of origin of the chia seed and also on the analytical method employed for quantification. The most abundant polyphenols in chia seeds include rosmarinic acid glucoside and rosmarinic acid, with 3.9 and 1.2 mg/g, respectively [45], and protocatechuic ethyl ester, with 0.74 mg/g [44] (Table 2). The antioxidant activity of the chia seed can be partly attributed to the presence of polyphenolic compounds that protect against cellular oxidative damage [46].

## 3. Therapeutic Properties of Chia Related to DM

### 3.1. Antihyperglycemic Effect 

A number of studies suggest that the flour, the oil, or the fractions derived from the chia seeds interfere with glucose metabolism. The possible benefits afforded by the incorporation of chia seed into the diet of Wistar rats, where chia was considered a substitute for the fat source in terms of dyslipidemia and insulin resistance, were investigated during periods ranging from 3 weeks to 2 months. After 3 weeks, it was observed that the incorporation of chia into the diet aided the prevention of the development of dyslipidemia and insulin resistance, while chia consumption for at least 2 months normalized both of these parameters [49]. 

Other investigations conducted by Enes et al., (2020) evaluated the in vivo effect of the flour and oil of chia seeds on glucose metabolism and insulin resistance. Chia seed flour and oil were administered to experimental groups of 10 rats that were fed with a high-fructose diet. The results showed that chia seed flour and chia seed oil not only modulated glucose metabolism but also decreased the adipose tissue content of the treated rats; moreover, chia seed oil improved tolerance to glucose and insulin and restored hepatocyte function. Additionally, these authors also investigated the in vitro effect of the hydrolyzed extract of phenolic compounds from chia seeds on glucose metabolism in a liver cell model. The results of this study suggested that the hydrolyzed extract of phenolic compounds gave rise to a decrease in the mRNA of the enzymes involved in gluconeogenesis through the control of AKT (a serine/threonine protein kinase) activation but also by means of the gluconeogenesis and glycolysis pathways [50]. 

On the other hand, Mihafu et al., investigated the effect on blood glucose, weight, or hematological parameters due to the incorporation of 20 g of chia flour or chia oil into the diet of rats. The treated rats exhibited a decrease in the postprandial glucose level, improved hematological parameters, and their weight gain was controlled. Additionally, higher levels of lipids were found in the feces of rats fed with chia seed flour or chia seed oil, indicating a slow digestion of fats due to the possible inhibition of the pancreatic lipase enzyme. These findings are interesting in that these authors propose the incorporation of chia seed into the diet as a promising alternative in primary prevention against T2DM and cardiovascular diseases [51]. As has been previously reported, adipose tissue dysfunction is considered a key factor in the development of diabetes; consequently, due to chia possessing a high fiber and omega-3 content, it could potentially improve glucose metabolism in individuals with problems related to adipose tissue, such as inflammation and the dysfunction of the pancreatic beta cells. Moreover, in patients with diabetes, chia favors a functional return of pancreatic cells, as well as a generally healthier adipose tissue state [52]. Then, another experiment was performed with diabetic rats fed a chia-enriched diet, in which it was observed that the final glucose levels were lower than those of the controls, supporting the probable relationship between the lipid profile and diabetes [53].

Fadwa et al., evaluated the antihyperglycemic and the lipid profile effect of a 100 mg/kg oral dose of chia seed aqueous extract in diabetic rats induced with Streptozotocin. A decrease in blood glucose, a reduction in total cholesterol level in plasma, and an increase in HDL were observed. In addition, the same aqueous extract exerted in vitro antioxidant activity through the uptake of the radical 2,2-diphenyl-1 picrylhydrazyl (DPPH) [54].

Investigations in healthy persons [55,56,57] and in patients with T2DM [58] revealed that the consumption of 7–24 g of the whole grain of chia seed incorporated into white bread or the ingestion of 34 g of whole-grain chia seed daily produced a reduction in the level of postprandial glucose and satiety in healthy individuals. In the case of patients with T2DM, the ingestion of chia seed gave rise to a decrease in blood pressure and improved coagulation, thereby reducing cardiovascular risk factors in this type of patient. 

At present, a diet high in fructose and one with a high fat content has been associated with the development of obesity, insulin resistance, and T2DM [59,60]. In this context, the effects of incorporating chia seed flour and chia seed oil into the diet of rats with a high fructose and high fat intake during 12 weeks were investigated. Changes in glucose tolerance, liver damage, and the expression of antioxidant enzymes were observed. As expected, the high-fructose diet induced insulin resistance, oxidative stress, and altered some obesity-related parameters. The incorporation of chia flour or chia oil into the diet improved glucose and insulin tolerance. Moreover, the chia seed oil caused an increase in the expression of the HSP70 and HSP25 proteins, while the chia seed only increased the expression of the HSP70. These proteins, called heat-shock proteins, promote responses for preventing cell damage in terms of various factors that cause stress or imbalance in homeostasis; the expression of these proteins is reduced in T2DM [61]. Furthermore, chia seed oil enhanced the expression of the antioxidant enzymes superoxide dismutase and glutathione peroxidase [62]. With these results, the authors presented an overview of the beneficial effects of chia, including chia flour and/or chia oil in the daily diet.

In contrast to the studies mentioned previously, a recent report found that a peptide fraction obtained from chia seed flour did not exhibit a hypoglycemic effect (postprandial glycemia) in Alloxan-induced diabetic rats administered with a single dose of 50 mg/kg [63].

### 3.2. Prevention of Risk of Cardiovascular Disease 

A substantial amount of evidence has shown that DM is closely related with coronary heart disease and cardiovascular events, due to its strong impact on the general vascular system. In this way, patients with DM are more susceptible to presenting myocardial infarction and death than persons without DM, this increasing the disease’s risk by up to 7 years in the elderly population. Consequently, cardiovascular disease in persons living with DM comprises an essential issue in the management of the disease, and prevention of the risk of cardiovascular disease comprises a necessary consideration for the detection and diagnosis of DM [64]. 

On the other hand, the beneficial effects of whole-grain intake in the daily diet for the prevention of cardiovascular diseases, such as coronary heart disease, the diminution of blood pressure, the decrease in the concentration of homocysteine, or even death, have been evidenced in previously published literature, in which dietary-fiber intake has been found to be inversely correlated with several cardiovascular disease risk factors. Therefore, in order to prevent cardiovascular disease, the recommendation of the increase in dietary fiber is supported by a variety of studies [65,66,67,68]. Furthermore, the modern and novel focus based on dietary schemes has demonstrated a positive effect on proinflammatory and prothrombotic markers, elevated blood pressure, and dyslipidemia, not only for the prevention of the risk of cardiovascular disease but also as complementary to current pharmacological treatments and as a therapeutic option in itself. In this regard, one of the most recognized dietary schemes is long-term supplementation of the whole grain of chia as an addition to conventional pharmacological therapy [58]. This clinical study included adult patients (aged 18–75 years) with type-2 DM (T2DM) controlled with oral therapy but in patients not taking insulin. The treatment consisted of the administration of chia as dietary supplementation in two forms: ground seeds and these seeds in breads that were especially manufactured for the study. After 12 weeks of treatment with chia, major cardiovascular risks revealed some benefits due the attenuation of systolic blood pressure (SBP) by 6.3 ± 4 mmHg (*p* < 0.001). In the case of the emerging risk factors, a reduction was observed in the high-sensitivity C-reactive protein (hs-CRP) (mg/L) by 40 ± 1.6% (*p* = 0.04) and in the von Willebrand factor (vWF) by 21 ± 0.3% (*p* = 0.03). Concerning the safety of chia in this study, no secondary effects were observed on coagulation, on liver enzymes, and on the kidney, even at the highest dose of n-3 PUFA considered in this study (37 g of chia). Thus, the results of this study showed that the administration of whole-grain chia as a supplement for the ordinary treatment of T2DM benefits the attenuation of the major and emerging cardiovascular risk factors without altering glycemic and lipid levels [58]. 

### 3.3. Inhibition of the Enzymes α-Glucosidase and α-Amylase

The enzymes α-glucosidase and α-amylase have demonstrated their successful role in plasma glucose regulation through the hypoglycemic effect due to their capacity to inhibit the assimilation of carbohydrates into the intestine. Furthermore, both of these enzymes have been useful in patients with early diabetes or combined with other drugs that are currently employed in clinical treatments of T2DM, such as the α-glucosidase inhibitors Acarbose (Precose^®^) and Miglitol (Glyset^®^). In recent decades, investigations have focused on the inhibitors of α-glucosidase and α-amylase of natural origin, especially from medicinal plants as alternatives or complements to the traditional pharmacological treatment for T2DM, due to the absence or to the slight side effects of affectations such as nausea, diarrhea, and abdominal pain [69]. Among these inhibitors deriving from natural sources, chia is highlighted due to its inhibitory effects on both enzymes. The peptide fractions obtained through the hydrolysis of chia seeds from the ultrafiltration method were the following: >10 kDa; 5–10 kDa; 3–5 kDa; 1–3 kDa; and <1 kDa. Among the latter, the largest fractions, that is, >10 kDa and 5–10 kDa, exhibited the highest inhibition on the α-amylase enzyme, with 85.61% and 79.19%, respectively. In contrast, the highest inhibition on the α-glucosidase enzyme (96.91%) was observed in the low-molecular-weight peptide fraction of >10 kDa. Even though products from other plant species exerted a higher inhibition than chia on both enzymes, that is, on α-glucosidase and α-amylase (IC_50_ = 5.34 mg/mL for α-glucosidase and IC_50_ = 121.46 mg/mL for α-amylase), as in the case of the grape seed extract or in that of purified anthocyanin from the *Berberis integerrima* fruit (IC_50_ = 0.71 ± 0.085 mg/mL for α-glucosidase and IC_50_ = 1.14 ± 0.003 mg/mL for α-amylase), chia can be considered the best option in terms of these plants because chia is an easily available source, is accepted by the worldwide population, and is without secondary effects, advantages that are not present in the other plants [69,70,71,72]. 

### 3.4. Anti-Inflammatory Effect

Inflammation occurs as a response to infection or injury; however, modern evidence has shown its relevance in diseases such as diabetes, cancer, and cardiovascular disease [73]. While there are various drugs that can counteract inflammation, the majority of these can also generate undesirable side effects; for these reasons, natural products and functional foods represent an attractive source of bioactive compounds with possible anti-inflammatory activity [74]. In this respect, the anti-inflammatory effect of mucilage (aqueous extract) and chia seed was evaluated in rats with arthritis, in rats with obesity, and in rats with obesity and arthritis. The experimental treatment was orally administered at doses of 100 mg of the dry mucilage/kg body weight (BW) or 300 mg oil/kg BW during 21 days. Rats with obesity and arthritis presented an increase in paw inflammation, in the level of plasma tumor necrosis factor-α (TNF- α), dyslipidemia, and oxidative stress. After the administration of chia products, both the mucilage and the chia oil gave rise to a decrease in the level of TNF-α, inflammation, and oxidative stress, as well as improving the lipid profile in obese and nonobese arthritic rats. These results suggest that chia seed oil and mucilage may be considered as nutraceuticals to combat the oxidative stress, lipid profile, and inflammation caused by rheumatoid arthritis [75].

Some other studies demonstrated, in in vitro and in vivo models, the anti-inflammatory activity of the peptide fractions of chia seed flour. Cárdenas et al., observed an inhibition produced by a protein fraction extracted at pH 3 on the denaturation of egg albumin [76]; this method is indicative of an inflammatory process, in that the denaturation of tissue proteins is one of the causes of inflammation [77].

The production of reactive species such as nitric oxide (NO) and H_2_O_2_ results as crucial for the inflammatory process. Thus, Chang et al., evaluated the anti-inflammatory activity of protein fractions obtained from chia seeds in the production of inflammatory process mediators. These peptide fractions caused a decrease in the release of NO and H_2_O_2_ in the cells and a diminution in the production of the superoxide radical, as well as in the expression of the enzyme superoxide dismutase or in inducible NO synthase. Therefore, as the peptide concentration increases, the mediators of the inflammatory process are interrupted. In the same manner, the low-molecular-weight protein fractions (1–3 KDa) gave rise to a decrease in the production of the cytokines involved in the stimulation of the inflammatory process, such as IL-1, IL-6, and TNF-α, and also detected an increase in cytokine IL-10, considered as anti-inflammatory. These low-molecular-weight protein fractions also demonstrated their ability to decrease TPA-induced edema in mouse ears, which may be due to decreased proinflammatory mediators, demonstrating the anti-inflammatory potential of chia seed protein fractions [78]. A similar anti-inflammatory effect was observed with the administration of chia seed in the food of rats with high-fructose diets, where an increase was recorded in the PPAR-γ protein (considered a negative regulator of inflammation) but a decrease in the level of TNF-α and an increase in antioxidant enzymes were also detected [79].

Because the anti-inflammatory process could be also mediated by the antioxidant capacity of polyphenols, the polyphenols identified in chia seeds, such as gallic acid, caffeic acid, chlorogenic acid, cinnamic acid, ferulic acid, quercetin, kaempferol, epicatechin, rutin, apigenin, and *p*-coumaric acid [80], have exhibited protective effects on the pancreatic beta cells, improving their regular functions [81]. Another of the main characteristics of the chia seed lies in the presence of omega-3 and omega-6 fatty acids, which are well described as anti-inflammatory in themselves [14,82].

### 3.5. Antioxidant Effect

In the genus *Salvia*, to which chia belongs, a wide range of polyphenolic antioxidants [83] and fatty acids [84] have been identified, in which these compounds naturally protect the seeds from both chemical and microbial decomposition. In this regard, it also has been considered that the antioxidant potential of chia seed is greater than that of the *Moringa oleifera* [85], reporting a major presence of flavonols, quercetin glycosides, kaempferol, omega-3 fatty acids [32], and gallic acid and its derivatives, all of these recognized as potent antioxidants [44]. The antioxidant potential of the chia seed impacts the improvement of various diseases such as hypertension, while the hydrolysate of the seed possesses excellent properties for eliminating free radicals [86]. A variety of in vitro chia seed assays focused on its antioxidant properties. Reyes-Caudillo and collaborators determined its antioxidant capacity by means of the ABTS radical scavenging assay, the β-carotene-linoleic acid model system (β-CLAMS), and the peroxidation of phospholipid liposomes [32]. The antioxidant activity of the chia seed extracts exerted a range of inhibition of ABTS^·+^ radical cation from 87.84 to 95.78%, comparable with the antioxidant capacity of the control Trolox^®^ with 96.60% radical cation inhibition. A similar antioxidant activity of chia seed was observed in the β-CLAMS assay and in lipid peroxidation, in which chia seed extracts demonstrated the same capacity to inhibit the β-carotene or lipid oxidation as Trolox^®^. The chia extracts, at 1% concentration, were effective as antioxidants in water-in-oil food emulsions, also showing a better capacity than the antioxidant control *tert*-Butylhydroquinone (TBHQ). Other antioxidant-assay determinations, such as the DPPH free-radical-scavenging assay and the ferric reducing antioxidant potential (FRAP) assay of the phenolic, flavonoid, and tannin extracts from chia seed, revealed that the flavonoid extract exhibited the highest antioxidant activity, with an IC_50_ value of 0.27 mg/mL in the DPPH assay and an EC_50_ of 0.06 mg/mL in the FRAP assay, where these results were comparable or even more potent that those exerted by the control [87]. 

In vivo studies demonstrated that the incorporation of chia into the diet generates antioxidant and beneficial effects in rats with chronic conditions such as diabetes. A diet with the addition of chia seeds and chia-seed oil for obese rats with high-fructose diets increased the activity of antioxidant enzymes, such as reduced glutathione (GSH), catalase (CAT), and glutathione peroxidase (GPx) in plasma, in a range of 35–47% compared to the control group [46]. Similarly, the implementation of chia in the diet of rats with high sucralose consumption or in ovariectomized rats induced the restoration of the activity of the antioxidant enzymes CAT, GPx, and superoxide dismutase (SOD), when compared to the control. Additionally, a decrease in the activity of the enzyme xanthine oxidase (XO), reactive oxygen species (ROS), and the levels of IL-6 and TNF-α were also detected in plasma [88,89]. Recent investigations reported that the quantity and quality of phenolic compounds obtained from chia seeds are essential for determining their antioxidant capacity; consequently, improvement in the extraction processes will, in turn, expand knowledge concerning the antioxidant activity of the chia seed [90,91]. This information confirms that the phenolic content in the different parts of the chia seed, as well as in the flour derived from it, enhances the possible application of the chia seed in dietary products, which would potentially retain the antioxidant activity for up to 1 year [76]. With this evidence, the chia seed becomes a promising ingredient for its incorporation into the diet and can improve oxidative stress processes and inflammation.

## 4. Discussion

DM is a multifactorial and multisystemic disease that, alarmingly, increases in terms of the number of cases of DM every year worldwide [1]. Currently, some of the investigations for developing drugs have focused on the search for agents that act on multiple targets or disease pathways to achieve the desired therapeutic effect. This approach represents an attractive option for the therapeutic treatment of DM, in which the focus is not only on controlling the glucose concentration (hypoglycemic effect) but also on other mechanisms that are involved in the decrease in the complications related with hyperglycemic conditions [92]. Among the latter, oxidative stress and inflammation are factors whose interactions have been demonstrated to play a key role in the pathogenesis and progression of DM, as well as in the development of DM complications. A number of recent studies revealed that inflammatory mediators such as IL-1β, IL-6, and TNF-α are related to DM. These mediators also favored the creation of ROS. The enormous productions of ROS and reactive nitrogen species (RNS) are closely linked with chronic inflammation as a result of a sustained long-term innate immune activation, which consequently can result in tissue damage. Certain other studies have reported that oxidative stress is closely linked with chronic hyperglycemic-induced insulin resistance. The chronic hyperglycemia that characterizes the DM condition facilitates the production of ROS and inflammation that could consequently also provoke cardiovascular fibrosis [93]. Extreme ROS formation, the reduction in the antioxidant defense mechanisms, and the increment of inflammation are directly related with the end-organ damage in DM. Thus, DM has been identified as the first cause of chronic renal failure, during which approximately >50% of end-stage renal disease requires replacement therapy such as dialysis or transplantation. In a similar manner, atherosclerosis is recognized as a macrovascular complication of DM related with accelerated endothelial injury. Recent evidence revealed that some inflammatory markers are related to atherosclerosis and kidney disease in DM by increasing their expression, such as vascular cell adhesion molecule-1 (VCAM-1), intracellular adhesion molecule 1 (ICAM-1), TNF-α, IL-1β, monocyte chemoattractant protein-1 (MCP-1), and NF-κB [94]. Thus, the release of inflammatory mediators is provoked by hyperglycemia, which, again, confirms the close interaction among DM, oxidative stress, and inflammation [95,96]. 

Despite the existence of several therapeutic agents for the treatment of DM, adverse effects have been observed, such as gastrointestinal disturbances, nausea, vomiting, hypoglycemia, and weight gain, and also the lack of effectiveness in some cases [97,98]. Nonetheless, the natural progression of DM calls for multiple drugs in addition to insulin. Therefore, multitarget drugs have been gaining interest as comprising a more effective approach in multifactorial diseases such as DM. Natural products derived from plants have proven interesting, since several of these exert a variety of activities that involve multiple targets in humans, such as the case of the bioflavonoid quercetin, which has been demonstrated to be active in muscles, pancreas, liver, and small intestine. The multitarget antidiabetic effect of quercetin involves its participation in the regulation of the glucose level through the skeletal muscles and also in the induction of adenosine monophosphate kinase (AMPK) activity in hepatocytes. Quercetin has also exhibited a protective effect on pancreatic beta cells from ROS and ameliorates the antioxidant defense status of the cells and tissues. Moreover, in addition to the antidiabetic properties of quercetin, this bioflavonoid has been proven to be a safe, neutralizable, and inexpensive product [97]. 

Other interesting natural products with multitarget antidiabetic actions include the xanthones isolated from *Swertia mussotii* (Gentianaceae), which have been used in traditional Tibetan medicine in the treatment of febrile hepatobiliary diseases. However, subsequent investigations have revealed that xanthones isolated from this species, such as mangiferin, bellidifolin, and methylswertianin, exhibited hypoglycemic properties [99,100]. Thus, 14 xanthones were evaluated in targets relevant for DM, such as antioxidant, aldose reductase (ALR2), and α-glucosidase. Xanthones as antioxidants and inhibitors of α-glucosidase were detected as follows: 1,3,7,8-tetrahydroxyxanthone; 1,3,5,8-tetrahydroxyxanthone; and 2,3,6,8-tetrahydroxyxanthone-7C-(b-d-glucoside), while 1,3,5,8-tetrahydroxyxanthone exhibited the most potent effect as an inhibitor of α-glucosidase (IC_50_ = 5.20.3 mm) and also as an inhibitor of ALR2 (IC_50_ = 88.61.6 mm). Due to the key role of ALR2 in the glucose metabolism of persons with DM, in which ALR2 participates in converting the excess of glucose into sorbitol, particularly in insulin-independent tissues such as nerve, lens, retina, and kidney, the compound 1,3,5,8-tetrahydroxyxanthone appears to be a candidate as a therapeutic agent not only in the control of DM but also in the complications associated with DM, such as neuropathies, nephropathies, and retinopathies [101]. In the case of the plant *Notholirion thomsonianum*, the methanolic crude extract was evaluated as an antidiabetic multitarget on the α-glucosidase and α-amylase enzymes, as well as an antioxidant in DPPH, ABTS, and H_2_O_2_ assays. The ethyl-acetate fraction demonstrated potent inhibition effects in all of the targets tested. The isolated Nt01-Nt03 compounds also exhibited activity on α-glucosidase, α-amylase, and as an antioxidant in the DPPH assay. Thus, *N. thomsonianum* represents a potential source of antidiabetic multitarget bioagents [102]. Although these natural products revealed antidiabetic and multitarget activities, chia may possibly be at an advantage in that it is an edible plant that possesses nutritional properties that benefit human health and, additionally, because of its acceptance and availability worldwide. 

The content of the fats present in chia has been importantly related with its health-promoting activities. Particularly, the ω-fatty acids have been recognized as responsible for the production of great health benefits. ω-3 fatty acids have demonstrated cardio-protective, anti-inflammatory, and hypotensive effects. Chia seeds are identified as the best source of ω-3 fatty acids due to their highest concentration of these among all of the available food sources. Therefore, many efforts to increase the functional value of foods have been focused on the addition of ω-3 fatty acids to foods [103]. Similarly, among the secondary metabolites present in chia that may be involved in its health-promoting activities, we mention ellagic acid due to its potent antioxidative and anti-inflammatory activities, especially in complications deriving from DM by the activation of multiple pathways such as radical production, the GPX/GSH system, NO/iNOS generation, AGE/ALE, JNK/ERK, and GLUT4. Moreover, recent evidence indicates the possible role of ellagic acid in the control of high glucose levels [104]. Likewise, chlorogenic acid participates in the protection against complications deriving from DM, such as diabetic nephropathy, diabetic retinopathy, and diabetic peripheral neuropathy. Furthermore, chlorogenic acid also exhibits effects such as hypoglycemic, hypolipidemic, anti-inflammatory, and antioxidant effects [105]. Apigenin exhibited a number of activities, such as inhibition of α-glucosidase, stimulation of insulin secretion, cardio-protective effects, and a potent antioxidant effect that activates the metabolism of glucose and its transportation through peripherical tissues [106]. During the last decade, quercetin has been identified as a promising metabolite in the treatment of DM due to its effects on the reduction in glucose levels, the increase in glucose tolerance, and the stimulation of pancreatic β-cells by the AMPK pathway in adipose tissue and muscles. Also, quercetin demonstrated an inhibitory effect on proinflammatory mediators, including TNF-α, IL-1, IL-4, IL-6, and IL-8, directly impacting on the protection of pancreatic β-cells [107]. 

On the other hand, genetic and environmental factors have been identified as important contributors to the development and progression of DM. Nevertheless, the alarming increase in the prevalence of DM reveals that the lifestyle observed in modern societies appears to be one of the factors of greatest impact that exert an influence on the appearance of DM due to dietary changes derived from the urbanization and economic growth having favored the consumption of unhealthy diets, which, in turn, include high caloric content, large portion sizes, abundant amounts of processed meat, highly refined carbohydrates, and saturated and trans fats. Thus, lifestyle factors including dietary ones are considered the keystones in DM prevention and treatment, and these are largely modifiable, representing a viable action that could target DM around the world [108,109,110].

The importance of individual nutrients, foods, and dietary patterns for the prevention and management of DM has been evident during recent decades through a number of clinical studies [110]. However, there is no definitive consensus concerning worldwide dietary habits, but a conventional and accepted diet for patients with DM comprises a balanced diet with continuous, moderate calorie restriction; notwithstanding this, patients exhibit difficulties in adhering to this diet [111]. Therefore, achieving a long-term diet in terms of food choices as well as of foods that can be versatile in their preparation represents an attractive option for successful adherence to the diet without compromising the overall quality of the diet and, even more so, these could enrich the diet in fiber or fatty acid content [110]. In this context, chia appears to be a solid candidate for inclusion in the recommended diet for the prevention and management of DM, mainly because of its nutritional properties and, particularly, due to its fiber content. The beneficial effects of the utilization of fiber are clearly determined in the reduction in the danger of cardiovascular disease and in diminishing the risk for developing T2DM in that the soluble fiber of chia reduces the absorption of glucose, lipids, and cholesterol in the small intestine, complying with the characteristics of a functional food that has been suggested for people with obesity, overweight, and T2DM [112]. In the same respect, the consumption of dietary fiber is directly related with the increase in satiety after meals and also with the decrease in the sensation of hunger later on. Then, due to the chia seed content of 34–40 g of dietary fiber per 100 g, representing the recommended daily intake for adults, the chia seed can be utilized in the treatment and prevention of diabetes [16]. In addition to having a high fiber content, chia seeds are also rich in PUFA such as linolenic acid (ω-3) and linoleic acid (ω-6) and in amino acids such as glutamic acid, arginine, and aspartic acid. These primary metabolites have been largely responsible for some of the most recognizable beneficial effects, in that chia seeds have shown to act directly on fat and glucose metabolism. In this way, linolenic acid has been ameliorating some metabolic disorders due to its effects on the storage of visceral fatty tissue, the modulation of the enzymes participating in lipolysis, the reduction in cardiac and hepatic inflammation, and the fibrosis that developed in rats because of a high-carbohydrate diet [113].

In 2009, the incorporation of chia seed as an ingredient in bakery products was authorized in the European Union. Later, this incorporation was extended to breakfast cereals and to mixtures of fruit, nuts, and seeds with a chia seed content of 5–10% [114]. Some clinical trials demonstrate that the consumption of foods (water, yogurt, and fruit juice) with 35 g of chia flour during 12 weeks in persons with hypertension produced a decrease in blood pressure [115]. Similarly, the consumption of white bread [55,56] or beverages [57] supplemented with chia seeds by healthy persons gave rise to a decrease in postprandial glucose levels. These studies present an encouraging picture, indicating that the addition of chia seed to food matrices does not interfere with the beneficial health effects of its primary and secondary metabolites, especially in the reduction in postprandial glucose and blood pressure. However, it is of interest to comply with the maximal recommended amount established in the regulations.

## 5. Conclusions

DM represents one of the main challenges in human health worldwide. Its metabolic complexity has led to the proposal of strategies that consider potential multitarget agents that may be more effective than only the hypoglycemic effect. In this regard, chia has exhibited properties, such as a hypoglycemic effect, as an inhibitor of α-glucosidase and α-amylase, as an anti-inflammatory, as an antioxidant, and in the prevention of cardiovascular disease. Furthermore, chia is widely recognized because of its nutrimental content, which exerts a beneficial impact on human health, especially due to its omega-3 fatty acids and its high concentration of dietary fiber. These aspects also permit chia to be proposed as a component of the diet for individuals living with DM. Therefore, chia may be considered an alternative in the treatment of DM with advantages over other natural products identified as antidiabetic multitargets due to its acceptance since pre-Hispanic times and the availability of chia as a component of the daily diet worldwide. However, more clinical trials that evaluate these antidiabetic multitargets in persons with DM are necessary. 

## Figures and Tables

**Table 1:** Nutrimental composition of chia seeds.

	USDA [1]	Other References
Energy/100 g of seed (kcal)	486	562 [18], 459.9 [19], 359.33–380.59 [15]
Protein (%)	16.5	24.2 [18], 19.6 [19], 24.6 [20], 18.18–19.72 [15]
Fat (%)	30.7	40.2 [18], 34.4 [19], 32.2 [20], 30.17–32.16 [15]
Carbohydrate (%)	42.1	26.9 [18], 17.7 [19], 26.2 [20], 2.23–4.59 [15]
Fiber (%)	34.4	30.2 [18], 23.7 [19], 33.37–37.18 [15], 34.5 [20]
Fatty acids, total saturated (%)	3.3	5.0 [18], 9.74 [19]
Fatty acid (%)		
14:0	0.03	0.06 [13,21], 0.03 [19]
15:0	0.03	0.04 [13], 0.03 [19]
16:0	2.17	7.10 [13], 7.04 [21], 6.69 [19]
17:0	0.063	0.06 [13,19]
18:0	0.912	3.24 [13], 2.84 [21], 2.67 [19]
20:0	0.093	0.24 [13], 0.02 [21], 0.09 [19]
22:0	0.03	0.08 [13]
Total monounsaturated fatty acids (%)	2.3	2.96 [18], 10.76 [19]
Fatty acid (%)		
14:1	0.03	
16:1	0.029	0.03 [21], 0.09 [19]
18:1	2.2	10.55 [19]
20:1	0.046	0.09 [19]
Fatty acids, total polyunsaturated (%)	23.7	22.8 [18], 79.47 [19]
Fatty acid (%)		
18:2	5.8	6.16 [18], 18.89 [21], 17.36 [19]
18:3	17.8	16.4 [18], 63.79 [21], 62.02 [19]
Amino acids (%)		
Tryptophan	0.436	
Threonine	0.709	0.54 [21]
Isoleucine	0.801	0.74 [21]
Leucine	1.37	1.42 [21]
Lysine	0.97	0.93 [21]
Methionine	0.588	0.67 [21]
Cystine	0.407	0.42 [21]
Phenylalanine	1.02	1.60 [21]
Tyrosine	0.563	0.61 [21]
Valine	0.95	0.79 [21]
Arginine	2.14	2.0 [21]
Histidine	0.531	0.61 [21]
Alanine	1.04	0.94 [21]
Aspartic acid	1.69	1.28 [21]
Glutamic acid	3.5	2.87 [21]
Glycine	0.943	0.91 [21]
Proline	0.776	1.28 [21]
Serine	1.05	0.94 [21]
Minerals (%)		
Calcium	63.1	45.60 [18]
Iron	0.77	0.92 [18]
Magnesium	33.5	44.90 [18]
Phosphorus	86.0	91.90 [18]
Potassium	40.7	72.60 [18]
Sodium	1.6	0.026 [18]
Zinc	0.46	0.647 [18]
Copper	0.09	0.186 [18]
Manganese	0.27	0.379 [18]
Selenium	5.52	0.004 [18]
Vitamins (%)		
Vitamin C	0.16	
Thiamin	0.062	
Riboflavin	0.017	
Niacin	0.88	
Vitamin A (IU/100 g of seed)	54	
Vitamin E	0.05	

**Table 2:** Secondary metabolites present in chia seed.

Compound	Concentration (µg/g)	Reference
Phenolic acids		
Gallic acid	11.5	[44]
	42.5	[47]
Caffeic acid	3.0–6.8	[32]
	27.4	[44]
	30.89	[19]
Chlorogenic acid	45.9–102.0	[32]
	4.68	[19]
	22.6–21.8	[48]
Protocatechuic ethyl ester	747.1	[44]
Rosmarinic acid	926.7	[44]
	1200	[45]
	635.98	[47]
Rosmarinic acid glucoside	3900	[45]
Dihydroxybenzoyl glucoside	189.6	[45]
Vanillic acid glucoside	165.7	[45]
Salvianic acid A	40.1	[45]
Fertaric acid	15.8	[45]
Phenols		
Oresbiusin A	15.0	[45]
Flavonoids		
Apigenin	0.005	[18]
	0.16–0.35	[15]
Kaempferol	360–509	[32]
	0.013	[18]
	24–25	[48]
Kaempferol 3-O-glucoside	0.028	[18]
Quercetin	0.17	[19]
	150–268	[32]
	285.56	[47]
	7–6	[48]
Myricetin	28.88	[47]
	115–121	[48]
Rutin	0.220	[18]
	99.88	[47]
Luteolin	5.91–15.79	[15]
Naringenin	0.22–0.39	[15]
Eriodictyol	4.17–8.95	[15]
Isoflavonoids		
Daidzein	457.85	[47]
	6.6	[44]
Glycitein	0.5	[44]
Genistein	55.69	[47]
	5.1	[44]
Genistin	19.58	[47]
	3.4	[44]
Catechin		
Epicatechin	0.0290	[18]

## Data Availability

Not applicable.
